# Advanced G‐CSF‐producing non‐small cell lung cancer‐not otherwise specified, with favourable response to pembrolizumab monotherapy

**DOI:** 10.1002/rcr2.625

**Published:** 2020-07-16

**Authors:** Yohei Matsui, Tadaaki Yamada, Naoko Masuzawa, Shinshichi Hamada, Koichi Takayama, Osamu Hiranuma

**Affiliations:** ^1^ Department of Pulmonary Medicine Otsu City Hospital Otsu Japan; ^2^ Department of Pulmonary Medicine, Graduate School of Medical Science Kyoto Prefectural University of Medicine Kyoto Japan; ^3^ Department of Pathology Otsu City Hospital Otsu Japan

**Keywords:** Biomarker, G‐CSF‐producing tumour, non‐small cell lung cancer‐not otherwise specified, pembrolizumab

## Abstract

Advanced granulocyte colony‐stimulating factor (G‐CSF)‐producing lung tumours are generally refractory to platinum‐based chemotherapy and are associated with poor prognosis. However, therapeutic strategies for these tumours remain unknown. A 74‐year‐old man was diagnosed with non‐small cell lung cancer‐not otherwise specified (NSCLC‐NOS); the clinical stage was T4N0M1c stage IVb. Blood testing showed leucocytosis and aberrant G‐CSF expression. We chose single‐agent pembrolizumab as the initial treatment because PD‐L1 was highly expressed in the tumours. A clinically favourable response was achieved from seven courses of pembrolizumab with a total disease‐free survival of 10 months. During this period, the blood leucocyte count was concordant with the disease condition. These observations showed that pembrolizumab monotherapy may be an effective treatment for patients with advanced G‐CSF‐producing NSCLC‐NOS and that the monitoring of leucocyte count may be a useful biomarker for predicting the efficacy of pembrolizumab monotherapy.

## Graphical abstract

Here, we report a patient with non‐small cell lung cancer‐not otherwise specified (NSCLC‐NOS) with high PD‐L1 expression and aberrant granulocyte colony‐stimulating factor (G‐CSF) production, in whom monotherapy with the anti‐PD‐1 antibody pembrolizumab was effective as an initial treatment.

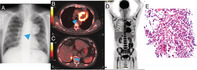

## Introduction

Granulocyte colony‐stimulating factor (G‐CSF) is a cytokine and hormone that stimulates the bone marrow to produce granulocytes and stem cells and release them into the bloodstream. Asano et al. demonstrated that G‐CSF was produced autonomously in the plasma of nude mice transplanted with human lung cancer cells [1]. G‐CSF‐producing tumours are clinically well‐characterized diseases with a poor prognosis. Of these, G‐CSF‐producing non‐small cell lung cancer‐not otherwise specified (NSCLC‐NOS) is an extremely rare and aggressive tumour that is refractory to platinum‐based chemotherapy, which is considered a standard treatment for patients with advanced NSCLC. Moreover, therapeutic strategies for this type of lung tumour remain unknown. Here, we report a patient with NSCLC‐NOS with high PD‐L1 expression and aberrant G‐CSF production, in whom monotherapy with the anti‐PD‐1 antibody pembrolizumab was effective as an initial treatment.

## Case Report

**Figure 1:**
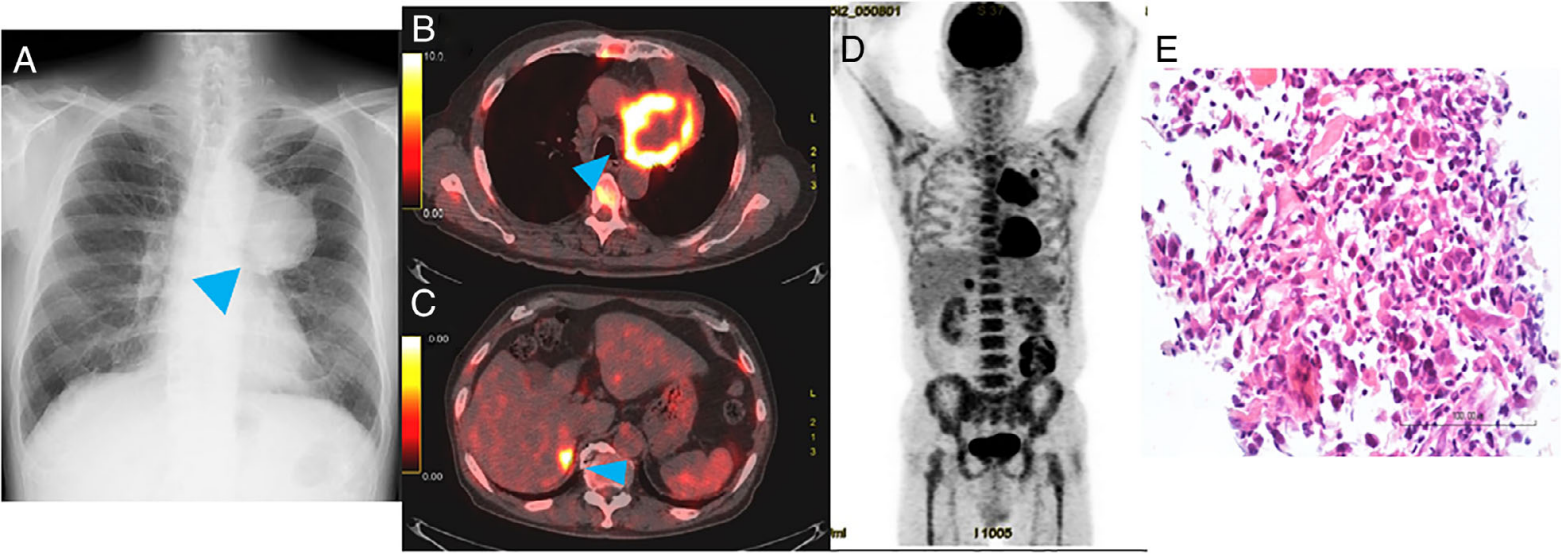
Image inspection on admission and pathological findings. (A) Chest radiograph showing a tumour shadow in the upper left lung field. (B–D) ^18^F‐fluoro‐d‐deoxyglucose (FDG) positron emission tomography‐computed tomography showed uptake of FDG in the left lung tumour (B), right adrenal gland (C), and bone marrow (D). (E) A biopsy specimen from the lung tumour. Higher magnification of Haematoxylin and eosin (HE) staining. Random proliferation of atypical epithelial cells with large, hyperchromatic nuclei are observed.

**Figure 2:**
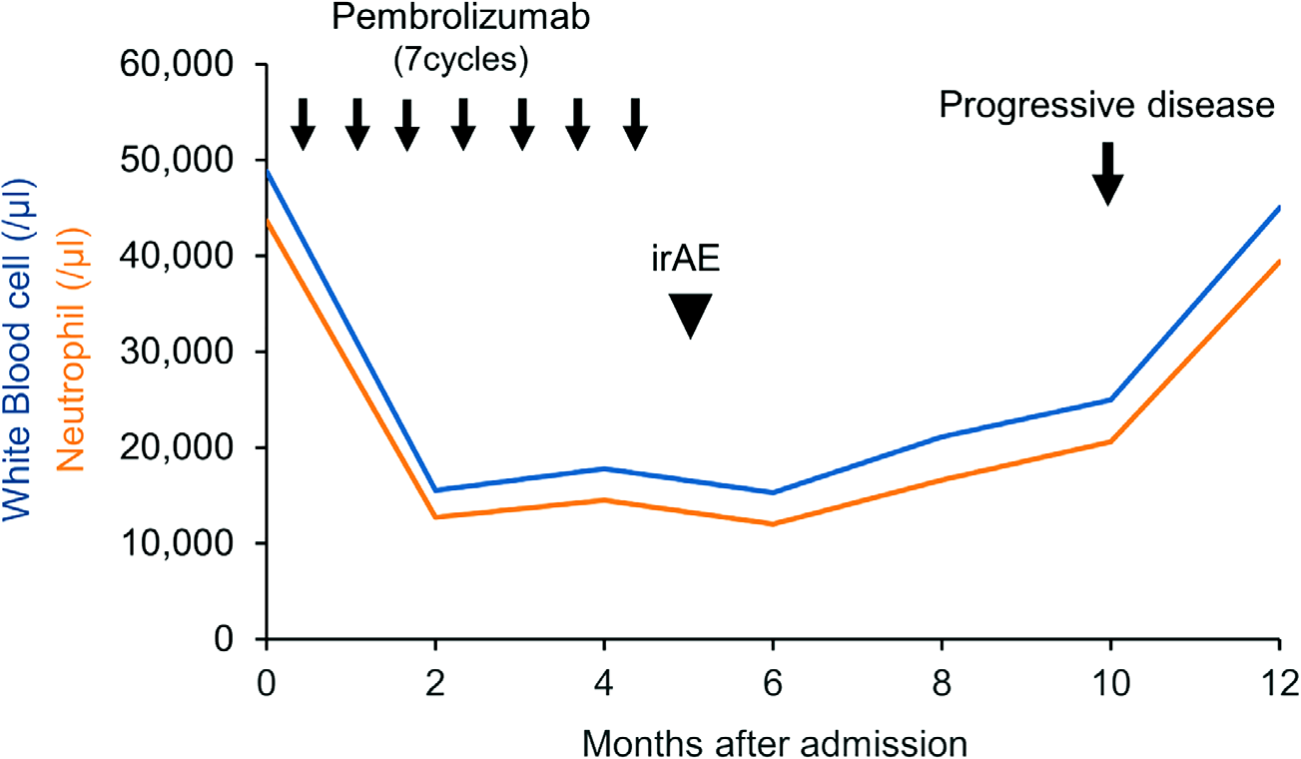
Clinical course of pembrolizumab monotherapy. Trends in the serum count of white blood cells and neutrophils are shown. irAE, immune‐related adverse event.

A 74‐year‐old man whose chest X‐ray findings indicated an upper left lung tumour was admitted to our hospital (Fig. 1A). He had smoked 30 pack‐years. His Eastern Cooperative Oncology Group performance status score was 0. The levels of squamous cell carcinoma antigen (SCC) and Salyl Lewis X‐i (SLX) were elevated to 2.2 ng/mL (normal range: 0–1.5 ng/mL) and 42.3 ng/mL (normal range: 0–38.0 ng/mL), respectively. Positron emission tomography‐computed tomography (PET‐CT) revealed fluorodeoxyglucose (FDG) uptake in a 6‐cm tumour shadow in the upper lobe of the left lung, metastatic tumours in the left lung and right adrenal gland, and a retroperitoneal tumour (Fig. 1B, C). In addition, increased diffuse bone marrow FDG uptake was observed (Fig. 1D). To confirm the diagnosis, a wedge resection of the left upper lung with the tumour shadow was performed, and the patient was diagnosed with T4N0M1c stage IVB primary NSCLC‐NOS (Fig. 1E), which was negative for thyroid transcription factor 1 (TTF‐1), napsin A, p40, chromogranin A, synaptophysin, and CD56 staining. The lung tumours showed a tumour proportion score (TPS) of 50–60% for programmed cell death ligand 1 (PD‐L1) (22C3) and no expression of epidermal growth factor receptor (EGFR) mutations and anaplastic lymphoma kinase (ALK) rearrangements. Blood testing on admission showed leucocytosis (48,800/μL) and neutrophilia (43,600/μL) with abnormally high expression of serum G‐CSF (502.2 pg/mL, normal range: <39.0 pg/mL). On the basis of these findings, the patient was diagnosed with advanced G‐CSF‐producing NSCLC‐NOS that was compatible with diffuse uptake of FDG into the bone marrow owing to G‐CSF‐producing carcinoma. We decided to treat him with a single agent, that is, the anti‐programmed cell death 1 (PD‐1) antibody pembrolizumab (200 mg/body) and repeated the treatment every three weeks because PD‐L1 was highly expressed in the tumours. The primary lesion and all metastatic lesions markedly shrank, and serum leucocytosis and neutrophilia were rapidly ameliorated, compared to the levels of tumour markers. After seven courses of pembrolizumab, grade 3 type 1 diabetes occurred, which was considered an immune‐related adverse event, and pembrolizumab treatment was discontinued. After that, the patient was disease‐free for five months, accompanied by a decrease in white blood cell (WBC) count and neutrophil count. At 10 months after the intervention with pembrolizumab monotherapy, leucocytosis and neutrophilia emerged; subsequently, relapsed tumours were confirmed by CT scan (Fig. 2). Eventually, the patient died 18 months after his first visit to our hospital.

## Discussion

The diagnostic criteria for a G‐CSF‐producing malignant tumour are: (1) marked increase in WBC count without infection or other diseases; (2) elevated serum G‐CSF level; (3) reduction in WBC count after tumour resection; and (4) G‐CSF‐positive staining from immunohistochemical analysis of the tumour [1]. In this case, leucocytosis and elevated serum G‐CSF activity without infection or other diseases were observed on admission. In addition, typical examination of G‐CSF‐producing tumours showed a diffuse accumulation of ^18^F‐FDG in the bone marrow in the PET scan, indicating glucose metabolism was enhanced with the promotion of haematopoietic ability [2]. Therefore, we clinically diagnosed the patient with GCF‐producing lung tumour. However, our limitation is that we could not evaluate the tumour by immunostaining for G‐CSF because of the strong necrosis with a few tumour cells.

Several previous case reports have shown the efficacy of systemic chemotherapy such as platinum‐combined chemotherapy with etoposide or paclitaxel and non‐platinum chemotherapy with gemcitabine and vinorelbine for G‐CSF‐producing lung tumours [3]. However, the standard chemotherapy for these type of lung tumours has not been established because most cases are aggressive and chemotherapy‐refractory with a poor prognosis. Therefore, it is necessary to develop novel systemic treatments.

Cancer immunotherapy has recently been developed as a promising alternative strategy for treating advanced NSCLC patients. In the KEY‐NOTE 024 clinical study, pembrolizumab monotherapy significantly prolonged progression‐free survival of NSCLC patients with high PD‐L1 expression, compared to systemic chemotherapy [4]. Therefore, pembrolizumab monotherapy is presently one of the recommended first‐line therapies for NSCLC patients with high PD‐L1 expression. This is the first report that shows beneficial disease control with pembrolizumab monotherapy as the first‐line treatment in a case of G‐CSF‐producing NSCLC‐NOS with high PD‐L1 expression.

Neutrophils induce pro‐inflammatory cytokines and chemokines that lead to tumour proliferation, invasion, and angiogenesis. However, there is no evidence on abnormal neutrophils by G‐CSF‐producing tumours having functional roles in the tumour microenvironment. Further investigations are warranted to elucidate these roles.

Serum WBC and neutrophil counts were more responsive to the abundance of tumour burden than tumour markers during immunotherapy and relapse of G‐CSF‐producing lung cancers. Therefore, monitoring leucocyte count may be a useful biomarker for predicting the efficacy of pembrolizumab monotherapy. Evidence accumulated from further cases are warranted to validate our observations.

### Disclosure Statement

Appropriate written informed consent was obtained for publication of this case report and accompanying images.
